# Supplementary material for: Effect of Temperature Cycling Pretreatment on the Thermal Stability of Sm2(Co, Fe, Zr, Cu)17 Magnets in the Mild Temperature Range
Source: Materials (Basel). 2022 Dec 10;15(24):8830. doi: 10.3390/ma15248830 (PMC9782894; doi:10.3390/ma15248830)
Supplement: Supplementary file 1 [file materials-15-08830-s001.zip › materials-1944973-supplementary.pdf]

materials

MDPI

Supplementary Materials

Effect of Temperature Cycling Pretreatment on the Thermal Stability of $Sm_2(Co, Fe, Zr, Cu)_{17}$ Magnets in the Mild Temperature Range

Hu-Lin Wu [1], Zhi-Mei Long [2], Zhong-Sheng Li [1]; Kai-Qiang Song [1], Chao-Qun Li [2], Da-Long Cong [1], Bin Shao [2,*], Xiao-Wei Liu [2], Jian-Chun Sun [2], and Yi-Long Ma [2]

[1] Southwest Institute of Technology and Engineering, Chongqing 400039, China; wuhulin59@163.com (W.H.-L.); zhongshli59@163.com (L.Z.-S.); scut_song@163.com (S.K.-Q.); congdl09@163.com (C.D.-L.)
[2] School of Metallurgy and Material Engineering, Chongqing University of Science and Technology, Chongqing 401331, China; 2021202024@cqust.edu.cn (L.Z.-M.); 2020202022@cqust.edu.cn (L.C.-Q.); lxwicq@163.com (L.X.-W.); Kwensun@163.com (S.J.-C.); yilongma@163.com (M.Y.-L.)
* Correspondence: shaobin19811107@163.com

Table S1. Temperature dependence of Φ and their losses of samples from R.T. to 750 °C.

Temperature/°C	Φ/mWb	Loss of Φ/%
Room Temperature	17.34	0.00
80	16.88	2.65
120	16.85	2.83
150	16.84	2.88
180	16.8	3.11
220	16.78	3.23
250	16.75	3.40
300	16.67	3.86
350	16.23	6.40
400	14.76	14.88
450	12.75	26.47
500	10.37	40.20
550	7.84	54.79
650	3.24	81.31
750	0.69	96.02

Table S2. Φ and their losses of samples cycled in the range of R.T. 180 °C and −50 °C — 180 °C.

Temperature range	Cycle number	Φ/mWb	Loss of Φ/%
R.T. — 180 °C	0	17.34	0.00
	10	17.21	0.75
	20	17.18	0.98
	40	17.15	1.04
	60	17.11	1.33
	80	17.08	1.52
	100	17.04	1.73
	125	17.05	1.67
−50 °C — 180 °C	0	17.08	0.00
	10	16.92	0.92

20	16.89	1.13
40	16.84	1.39
60	16.82	1.50
80	16.78	1.76
100	16.75	1.95
125	16.72	2.13

Table S3. Values of fitting parameters and their standard errors (*S.E.*) in the different temperature range. The fitting formulae are given by $Y=A\times(X-Xc)^P$.

Y	**Temperature range**	**Fitting Parameter**						
		X_c	$S.E._{(Xc)}$	A	$S.E._{(A)}$	P	$S.E._{(P)}$	R^2
**Loss of *Φ***	R.T. — 180 °C	0.00	10.49	0.34	0.18	0.33	0.11	0.97
	−50 °C — 180 °C	0.00	6.34	0.42	0.13	0.33	0.06	0.99

Table S4. The *Φ* and their losses of samples recycled in the range of R.T. to 180, 250, and 300 °C, respectively.

**Temperature range**	**Cycle number / -**	***Φ* / mWb**	**Loss of *Φ* /%**
R.T.–180 °C	0	17.34	0.00
	10	17.21	0.75
	20	17.18	0.98
	40	17.15	1.04
	60	17.11	1.33
	80	17.08	1.52
	100	17.04	1.73
	125	17.05	1.67
R.T. — 250 °C	0	17.84	0.00
	10	17.67	0.95
	20	17.58	1.48
	40	17.57	1.53
	60	17.51	1.85
	80	17.48	2.00
	100	17.45	2.17
	125	17.43	2.30
R.T.–300 °C	0	17.19	0.00
	10	16.98	1.24
	20	16.91	1.65
	40	16.85	2.00
	60	16.79	2.31
	80	16.76	2.48
	100	16.74	2.64
	125	16.70	2.85

Table S5. The *Φ* and their losses of samples hold in air at 80, 120, and 180 °C for different days, respectively.

**Constant temperature/°C**	**Holding time/Day**	**Untreated**		**Pretreatment: −50 °C–250 °C × 3 cycles**	
		***Φ*/mWb**	**Loss of *Φ*/%**	***Φ*/mWb**	**Loss of *Φ*/%**
80 °C	original	/	/	17.16	0.00
	0	17.20	0.00	16.90	1.40
	22	17.01	1.47	16.82	1.90

	45	16.93	1.91	16.80	2.01
	60	16.89	2.12	16.79	2.07
	90	16.84	2.43	16.78	2.10
	120	16.81	2.59	16.76	2.25
	150	16.81	2.63	16.74	2.33
	180	16.79	2.72	16.72	2.43
	original	/	/	17.16	0.00
	0	17.26	0.00	16.89	1.46
	22	17.01	1.85	16.82	1.95
	45	16.91	2.42	16.79	2.08
120 °C	60	16.86	2.71	16.77	2.20
	90	16.85	2.75	16.75	2.31
	120	16.84	2.83	16.74	2.41
	150	16.80	3.04	16.72	2.51
	180	16.78	3.17	16.70	2.56
	original	/	/	17.19	0.00
	0	17.33	0.00	16.89	1.63
	22	16.98	2.06	16.82	2.15
	45	16.86	2.71	16.79	2.36
180 °C	60	16.83	2.88	16.77	2.47
	90	16.81	3.04	16.76	2.53
	120	16.80	3.06	16.74	2.62
	150	16.77	3.23	16.73	2.70
	180	16.75	3.38	16.70	2.79

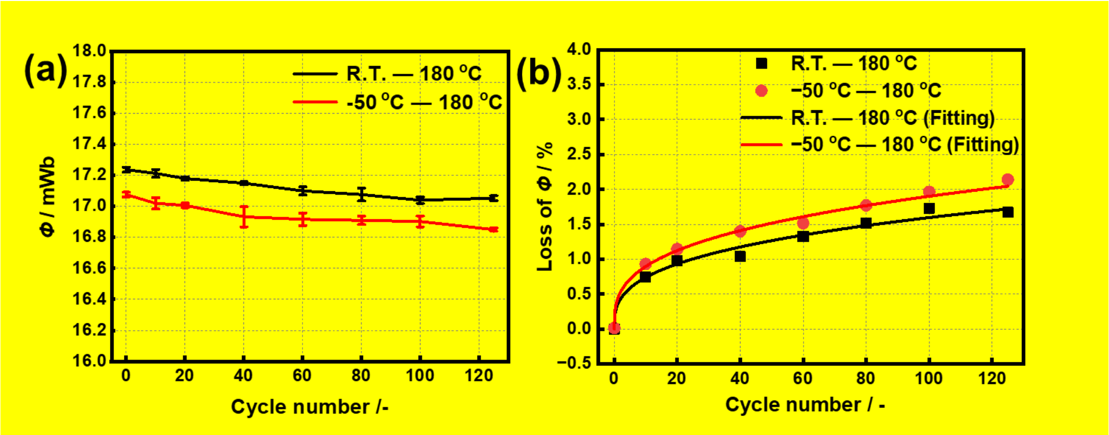

Figure S1. Variation trends of (**a**) Φ, (**b**) The fitting curve of the loss of Φ during recycling between the R.T. and 180 °C, and between the −50 °C and 180 °C for 125 cycles, respectively.

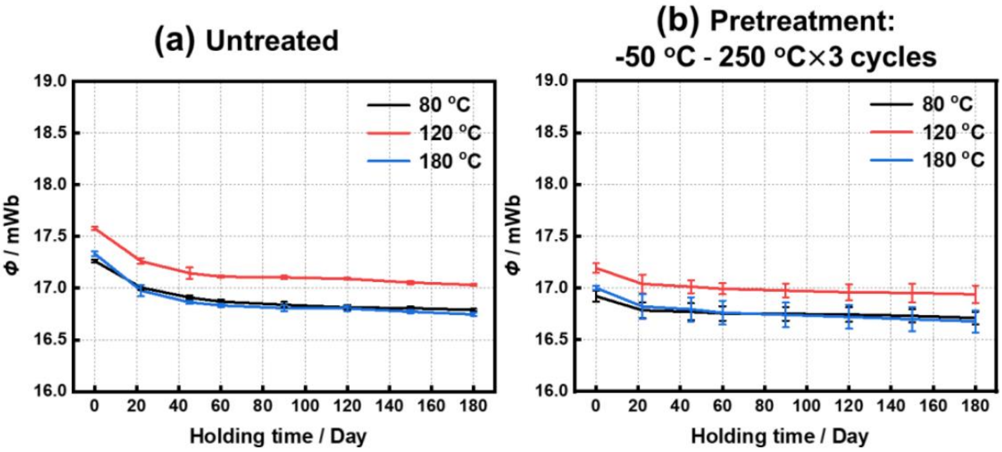

Figure S2. Variation trends of Φ of (**a**) untreated and (**b**) pretreated samples held in air at 80, 120, and 180 °C for 180 days, respectively.
